# Dexmedetomidine Co-Administered with Lidocaine Decreases Nociceptive Responses and Trigeminal Fos Expression without Motor Dysfunction and Hypotension in a Murine Orofacial Formalin Model

**DOI:** 10.3390/life12020215

**Published:** 2022-01-30

**Authors:** Ji-Hee Yeo, Dae-Hyun Roh

**Affiliations:** Department of Oral Physiology, School of Dentistry, Kyung Hee University, Seoul 02447, Korea; duo9427@naver.com

**Keywords:** dexmedetomidine, lidocaine, mice, nociception, rotarod performance test, trigeminal nuclei, blood pressure, adrenergic agonists

## Abstract

Administration of dexmedetomidine significantly induces sedation and anti-nociception in several nociceptive models, but clinical trials are restricted due to adverse side effects, including lethargy, hypotension, and bradycardia. Herein, we investigated whether intraperitoneal inoculation of dexmedetomidine reduced the orofacial nociceptive response and affected motor coordination and blood pressure and examined whether a lower dose of dexmedetomidine in combination with 0.5% lidocaine produced an antinociceptive effect without any adverse side events in a murine model. To perform the experiment, 5% formalin (10 µL) was subcutaneously inoculated into the right upper lip, and the rubbing responses were counted for 45 min. Different doses of dexmedetomidine combined with 0.5% lidocaine were administered 10 and 30 min before formalin injection, respectively. Dexmedetomidine (10 μg/kg) significantly reduced orofacial nociceptive responses during the second phase of the formalin test and decreased the expression of Fos in trigeminal nucleus caudalis (TNC). Besides, a high dose of dexmedetomidine (30 μg/kg) induced lessening physical ability and significantly reduced systolic pressure and heart rate. When 0.5% lidocaine was injected subcutaneously, nociceptive responses were reduced only in the first phase. Interestingly, although a low dose of dexmedetomidine (3 μg/kg) alone did not show an antinociceptive effect, its co-administration with lidocaine significantly reduced the nociceptive response in both phases and decreased TNC Fos expression without motor dysfunction and hypotension. This finding suggests that the combination of a low-dose of systemic dexmedetomidine with lidocaine may be a safe medicinal approach for acute inflammatory pain management in the orofacial region, particularly mucogingival pain.

## 1. Introduction

Orofacial pain (OFP) disorders are highly prevalent and debilitating conditions can arise from different regions and etiologies, including pathological conditions of the teeth and related structures [1]. Besides, these conditions represent a real challenge in the clinic since the orofacial region is complex. Therefore, the clinician must have a solid awareness and insight of the pain mechanisms [2,3] that develop from these structures for proper diagnosis; a multidisciplinary approach for OFP management is strongly recommended.

Dexmedetomidine, a potent α2-adrenoceptor agonist, was first authorized to be used as a sedative agent in the intensive care unit. In addition, dexmedetomidine was used as an adjuvant for pain treatment, mostly during the acute perioperative settings [4]. In animal studies, dexmedetomidine induced an antinociception effect at the spinal cord level revealed by the tail-flick tests [5] and significantly attenuated both nociception and hyperalgesia in acute and chronic pain rodent models [6,7]. Moreover, Nazarian et al. reported that dexmedetomidine attenuated intraplantar formalin-induced Fos-protein expression in the ipsilateral superficial dorsal horn [8]. Although several studies have proven the analgesic effect of dexmedetomidine, the improvement of nociception and hyperalgesia by the activation of the α2-adrenoreceptor with dexmedetomidine in the orofacial region remains unclear. Furthermore, the common side effects of dexmedetomidine, such as impaired motor function, hypotension, and bradycardia, have been a common problem with the currently available α2-adrenoceptor agonists after either systemic or spinal administration [9].

Lidocaine, a local anesthetic used in surgery and dentistry, can be administered in multiple methods, most often as a nerve block or infiltration, depending on the nature of care performed and the extent of the mouth being worked on [10]. The pharmacological action of lidocaine leads to block the neuronal signal conduction by inactivating the voltage-gated Na^+^ channels in the neuronal cell membrane [11]. Thus, its local administration produces antinociceptive effects both in acute postoperative and chronic neuropathic pain [12].

To date, local administration of dexmedetomidine has been focused on several clinical studies [13,14,15,16]. On the other hand, systemic injection of dexmedetomidine (intraperitoneal, i.p. or intravenous, i.v.) has been widely used as a sedative and, in particular, several studies have reported that dexmedetomidine could be used for both non-invasive and invasive procedural sedation in infants and children [17,18,19]. Therefore, we considered that the systemic treatment of a lower dose of dexmedetomidine, which may induce analgesic effects, but not sedation, is worthy of basic and clinical research in the fields of pediatric dentistry and dentistry for the disabled with OFP. In this study, we aimed at assessing whether intraperitoneal injection of dexmedetomidine inhibits the facial nociceptive responses and decreases the Fos-protein expression in the trigeminal nucleus caudalis (TNC) region using a mouse orofacial formalin model. Moreover, we aimed at investigating whether the co-administration of dexmedetomidine with local anesthetic lidocaine could potentiate its antinociceptive effects without creating any adverse effects, including impaired motor coordination and a decrease in systolic blood pressure (SBP) and heart rate.

## 2. Materials and Methods

### 2.1. Animal

Male C57BL/6 mice (25–30 g, DBL Co., Seoul, Korea), housed in colony cages, were allowed free access to food and water and were retained in the animal facility, which was on a 12-h light/dark cycle, held at constant temperature (21–25 °C), and humidity (45–50%) until the day of the experiment. All experimental procedures were in accordance with the National Institutes of Health Guide for the Care and Use of Laboratory Animals and approved by the Institutional Animal Care and Use at the Kyung Hee University [KHUASP(SE)-16-014].

### 2.2. Drugs

Dexmedetomidine hydrochloride (Tocris, Bristol, UK) was dissolved in physiological saline as a stock solution of 10 mg/211.2 μL and was then diluted in physiological saline at doses of 3, 10, and 30 μg/kg. Lidocaine 2% (20 mg/mL, Huons, Seongnam, Korea) was diluted in physiological saline to get the final concentration of 0.5% (5 mg/mL). Dexmedetomidine was inoculated i.p. 30 min before formalin injection and co-administered with 0.5% lidocaine subcutaneously (s.c.) into the right upper lip 20 min after dexmedetomidine injection. All experiments were performed as blind tests.

### 2.3. Formalin-Induced Orofacial Pain Test

**Figure 1:**
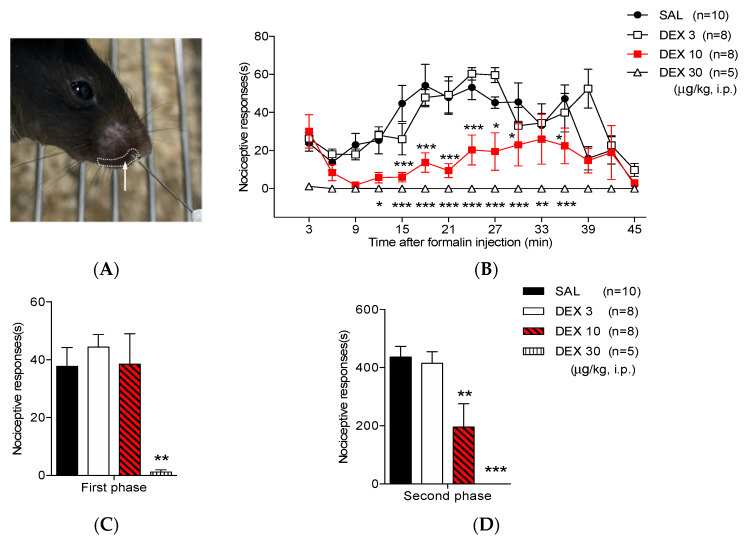
Effect of dexmedetomidine (DEX) in the orofacial formalin test in mice. (**A**) Injection site of the formalin solution. (**B**) 3 μg/kg of DEX did not restrain orofacial formalin-induced nociceptive responses. Conversely, medium- and high dose of DEX (10 and 30 μg/kg, respectively) significantly diminished nociceptive responses at 15–27 and 0–45 min after formalin injection, respectively. Medium- and high dose of DEX (10 and 30 μg/kg) revealed potent antinociceptive effects in the (**C**) first and (**D**) second phases compared with that in the saline (SAL)-treated group (* *p* < 0.05, ** *p* < 0.01, and *** *p* < 0.001 as compared with that in the SAL group, *n* = 5–10 per group).

The orofacial formalin test, previously described [20,21], was performed using male C57BL/c mice that were acclimatized for 30 min in an acrylic observation chamber (15 × 15 × 15 cm) and inoculated with 10 µL of 5% formalin s.c. using a 30-gauge needle attached to a Hamilton syringe into the right upper lip, lateral to the nose (Figure 1A). Animals were immediately placed back into the observation chamber; nociceptive responses were recorded for 45 min in each animal using a video camera; a nociceptive score was established for each block (3 min each) by measuring the number of seconds the animal spent cleaning the injected area with the ipsilateral forepaw. The face wiping or rubbing behavior with the forepaw was counted as a nociceptive response, while the scratching behavior with the hind paw was excluded, as it was considered an itch reaction [22]. The duration of the responses during the first two blocks represented the first phase (0–6 min postinjection), whereas the duration of responses during the subsequent 13 blocks represented the second phase (6–45 min postinjection) in the formalin-induced OFP test.

### 2.4. Fos Immunohistochemistry

TNC Fos immunohistochemistry was conducted as previously described [21]. The animals used in pain behavioral tests were used for Fos immunohistochemistry study. To minimize animal sacrifice, the minimum number of animals required for statistical analysis (*n* = 5–6) was randomly selected. Two hours after the formalin injection, animals were deeply anesthetized with 5% isoflurane and transcardially perfused through the ascending aorta with 50 mL of 0.1 M phosphate-buffered saline (PBS) at pH 7.4, followed by 4% paraformaldehyde. After perfusion, the brainstem was immediately removed, stored at 4 °C overnight in the same fixative, and then placed in a cryoprotectant solution (30% sucrose in PBS) for at least two nights at 4 °C before sectioning. Serial transverse sections (30 µm) from the TNC were obtained using a cryostat (Leica Microsystems, Wetzlar, Germany) and collected in PBS. Endogenous peroxidase activity was eliminated using 3% hydrogen peroxide diluted in PBS and tissues were pre-blocked with 3% normal goat serum and 0.3% Triton X-100 in PBS. Sections were incubated with polyclonal rabbit anti-Fos antibody (1:1000 Santa Cruz Biotechnology Inc., Santa Cruz, CA, USA) overnight at 4 °C. After several PBS washes, tissue sections were incubated with a secondary biotinylated anti-rabbit antibody (1:200, Vector Laboratories, Burlingame, CA, USA) for 1 h at room temperature and were processed using the avidin-biotin method (Elite ABC; Vector Laboratories). Fos-immunoreactive (ir) cells were visualized using a 3-3-diaminobenzidine reaction intensified with 0.2% nickel chloride.

### 2.5. Image Analysis

The TNC tissue sections were scanned using the brightfield and fluorescent microscope ECLIPSE 80i (Nikon Corp., Kanagawa, Japan) and digitized using a cooled CCD camera (Cool Snap ES model, Nihon Roper, Tokyo, Japan). Six nonadjacent tissue sections per mouse were randomly selected and quantitatively analyzed using a computer-assisted image analysis system (MetaMorph version 7.7.2.0, Westchester, PA, USA). The shape factor was set to a range of 0.5 to 1.0 and Fos-ir cells were counted only if they were at least 30% darker than the average gray level of each image [23]. The average number of Fos-ir cells was obtained per section from each animal. These values were averaged across each group and all analytical procedures described above were blindly performed without knowing in advance the experimental conditions.

### 2.6. Rota-Rod Test

The rotarod test, commonly used to detect motor ataxia in rodents, was used in this study to assess the potential sedative effects of dexmedetomidine. Briefly, mice were placed on the horizontal bar with a rotation speed of four revolutions per minute. All mice were tested 24 h before the actual rotarod test and those that stayed on the rod for at least 120 s were included in the study. Thirty minutes after dexmedetomidine injection, each animal was subsequently tested on the rotarod over a 2 min period, and their performance time on the bar (in seconds) and the number of falls were quantified. The test was repeated three times consecutively and the mean value for each animal was documented.

### 2.7. Assessment of Systolic Pressure and Heartbeat

SBP and heart rate were also assessed using a noninvasive computerized tail-cuff system (PowerLab system; ADI Instrument Pry Ltd., Chain Hills, NSW, Australia) as previously described [24]. Briefly, animals were acclimated for 1 h in a quiet test room, and the SBP and heart rate were assessed. Each experiment was repeated three times, and the mean value for each animal was recorded. SBP and heart rate were recorded 5 min (PRE) before and 30 min (POST) after dexmedetomidine injection.

### 2.8. Statistical Analysis

All values are expressed as mean ± standard error of the mean (S.E.M). Either two-way repeated-measures or one-way ANOVA followed by a posthoc Bonferroni test was conducted for multiple comparisons in the formalin pain behavior test. Fos immunohistochemistry, rotarod test, and SBP data were analyzed using one-way ANOVA, followed by a post hoc Bonferroni test. All statistical analyses were performed using GraphPad Prism (Version 6.0, GraphPad Software, San Diego, CA, USA) and all *p*-values of <0.05 were considered statistically significant.

## 3. Results

### 3.1. Dexmedetomidine Reduces Orofacial Formalin-Induced Nociceptive Responses

Mice injected with saline (SAL) i.p. and orofacial 5% formalin exhibited typical biphasic pain behaviors during the 45 min observation period (phases 1 and 2). The injection of low-dose dexmedetomidine (3 μg/kg) did not show an antinociceptive effect in orofacial formalin-induced nociceptive responses as compared with the SAL-treated group. Conversely, the nociceptive responses in mice treated with 10 μg/kg of dexmedetomidine significantly decreased at 15–30 and 36 min after formalin injection as compared with the SAL-treated group. Additionally, a high dose of dexmedetomidine (30 μg/kg) seemed to reduce orofacial nociceptive responses at 12–36 min as compared to the SAL-treated group (Figure 1B) but was considered a sedative effect because voluntary movement did not occur in all mice treated with dexmedetomidine 30 μg/kg.

The total of orofacial formalin-induced nociceptive behavior time in the SAL-treated group was 37.77 ± 6.49 s and 436.31 ± 36.81 s during the first and second phases, respectively. Pretreatment with 10 μg/kg of dexmedetomidine reduced the sum of formalin-induced nociceptive responses during the second phase, whereas a high dose of dexmedetomidine (30 μg/kg) appeared to induce a potent sedation rather than antinociception during both phases. (Figure 1C,D and Table 1).

### 3.2. Dexmedetomidine Reduces the Increase of Fos-ir Cells in TNC

**Figure 2:**
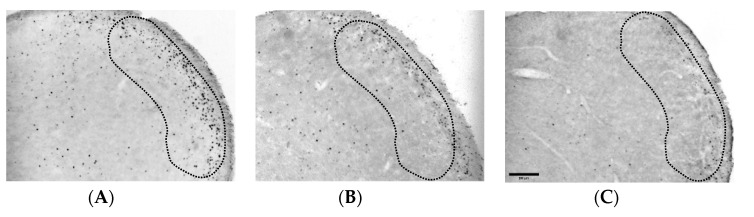

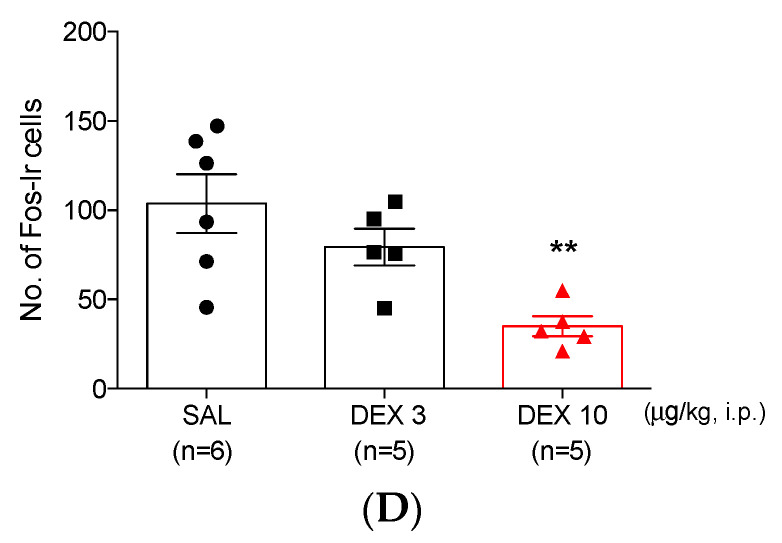
The outcome of dexmedetomidine (DEX) on the Fos-ir cells in the trigeminal nucleus caudalis (TNC). The number of Fos-ir cells increased in the TNC of the (**A**,**D**) SAL-treated group; (**B**,**D**) 3 μg/kg of DEX did not defeat the increase of Fos-ir cells. Conversely, (**C**,**D**) a medium dose of DEX (10 μg/kg) significantly reduced the increase of Fos-ir cells in the ipsilateral TNC (** *p* < 0.05 as compared with that in the SAL group, *n* = 5–6 per group). The boundaries of the TNC are outlined by the dotted line. Scale bar = 200 µm.

The number of Fos-ir cells was 103.69 ± 16.48 in the TNC of the SAL-treated group (Figure 2A,D). Although a low dose of dexmedetomidine (3 μg/kg) did not decrease the number of Fos-ir cells (79.35 ± 10.24; Figure 2B,D), 10 μg/kg of dexmedetomidine had significantly suppressed the number of Fos expression (34.99 ± 5.64) in the TNC as compared with those in the SAL-treated group (Figure 2C,D).

### 3.3. High-Dose Dexmedetomidine Induces Motor Dysfunction and Decreases Both Systolic Pressure and Heart Rate

**Figure 3:**
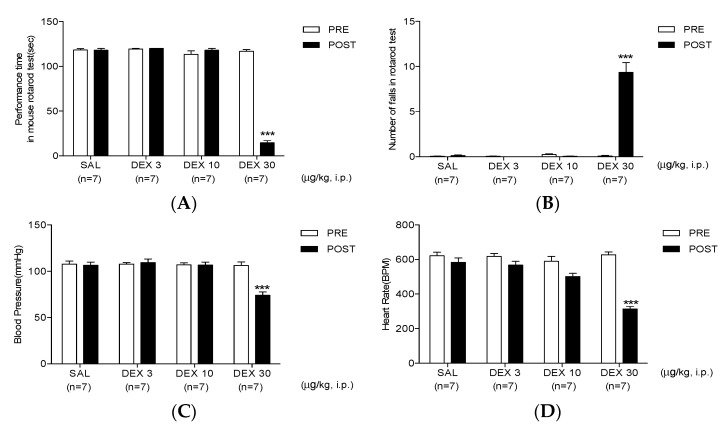
Effect of dexmedetomidine (DEX) on motor function and systolic blood pressure and heart rate. High-dose DEX (30 μg/kg) lessened the (**A**) performance time, increased (**B**) the number of falls and caused a significant drop in (**C**) systolic blood pressure and (**D**) heart rate (*** *p* < 0.001 as compared with that in the SAL group, *n* = 7 per group). Low- and medium-dose DEX (3 and 10 μg/kg) had no effect on motor coordination and blood pressure.

The rotarod test revealed that both SAL or dexmedetomidine (3 and 10 μg/kg) injection did not affect motor coordination. Conversely, 30 μg/kg of dexmedetomidine, inoculated intraperitoneally, significantly decreased the performance time and increased the number of falls (Figure 3A,B).

Similar results were observed during the assessment of SBP and heart rate, where SAL or 3 and 10 μg/kg of dexmedetomidine injection did not affect the normal blood pressure and heart rate. Conversely, 30 μg/kg of dexmedetomidine caused a significant decrease in SBP and heart rate (74.01 ± 3.57 and 313.95 ± 14.66, respectively; Figure 3C,D).

### 3.4. Co-Administration of Dexmedetomidine with 0.5% Lidocaine Reduces Orofacial Formalin-Induced Nociceptive Responses

**Figure 4:**
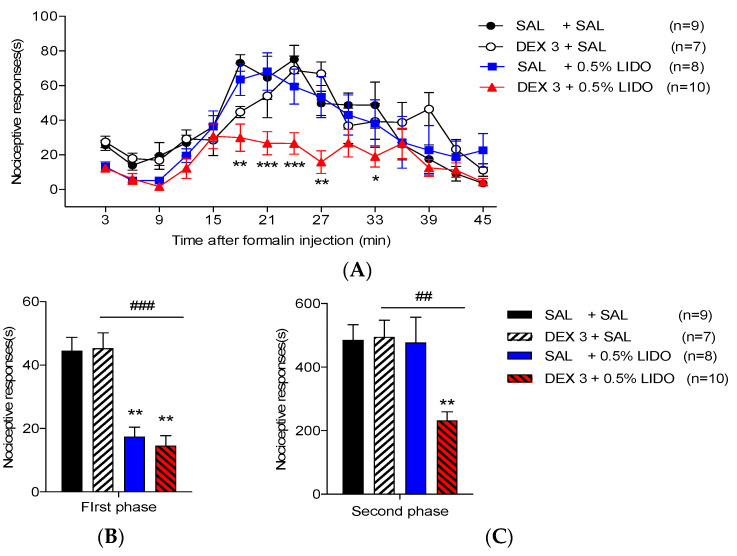
Influence of co-administration of 0.5% lidocaine with saline (SAL) or dexmedetomidine (DEX) in the orofacial formalin test in mice. (**A**) Co-administration of 0.5% lidocaine with SAL did not inhibit the orofacial formalin-induced nociceptive response. (**B**) However, co-administration of 0.5% lidocaine with SAL reduced the nociceptive responses only in the first phase (** *p* < 0.05 as compared with that in the SAL + SAL group, *n* = 8–9 per group). (**A**) Conversely, co-administration of 0.5% lidocaine with an ineffective lower dose of DEX (3 μg/kg) was significantly lowered 15–27 and 33 min after formalin injection. (**B**,**C**) Additionally, co-administration of 0.5% lidocaine with low-dose DEX (3 μg/kg) produced a more potent antinociceptive effect during both phases (* *p* < 0.05, ** *p* < 0.01, and *** *p* < 0.001 as compared with that in the SAL + SAL group and ^##^ *p* < 0.01, ^###^ *p* < 0.001 as compared with that in the DEX 3 + SAL group, *n* = 7–10 per group).

The effect of 0.5% lidocaine in orofacial formalin-induced pain has been evaluated. Statistically, a significant difference in the time course of nociceptive responses was not found when the co-administration of SAL with 0.5% lidocaine (SAL + 0.5% LIDO) group was compared with that in the SAL + SAL group (Figure 4A). Conversely. there was a significant decrease in nociceptive responses during the first phase in the SAL + 0.5% LIDO group (17.47 ± 2.97 s; Figure 4B and Table 2). This decrease was not observed during the second phase (Figure 4C and Table 2).

Taking into consideration the previous results, the co-administration of 0.5% lidocaine with an ineffective lower dose of dexmedetomidine (3 μg/kg) generated a synergistic antinociceptive effect has been subsequently investigated. Co-administration of 0.5% lidocaine with 3 μg/kg of dexmedetomidine significantly decreased nociceptive responses at 18–27 and 33 min after formalin injection as compared with that in the SAL + SAL group (Figure 4A). Moreover, co-administration of 0.5% lidocaine with 3 μg/kg of dexmedetomidine triggered an antinociceptive effect in both phases (Figure 4B,C and Table 2).

### 3.5. Co-Administration of Dexmedetomidine with 0.5% Lidocaine Reduces Fos-ir Cells

**Figure 5:**
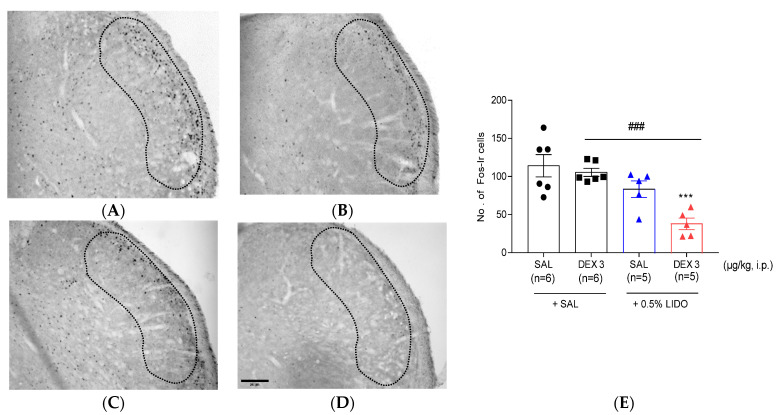
Effect of co-administration 0.5% lidocaine with dexmedetomidine (DEX) on the rise of Fos-ir cells in the trigeminal nucleus caudalis (TNC). (**A**–**C**,**E**) Either the co-administration of 3 μg/kg of dexmedetomidine with SAL or the co-adiminsration of 0.5% lidocaine with saline (SAL) did not suppress Fos expression after orofacial formalin injection. (**D**,**E**) Conversely, co-administration of 0.5% lidocaine with the ineffective lower dose of DEX (3 μg/kg) significantly diminished the increase of Fos-ir cells in the ipsilateral TNC (*** *p* < 0.001 as compared with that in the SAL + SAL group and ^###^ *p* < 0.001 as compared with that in the DEX 3 + SAL, *n* = 5–6 per group).

Figure 5 shows the effect of co-administration of dexmedetomidine with 0.5% lidocaine in TNC Fos expression. Either the co-administration of 3 μg/kg of dexmedetomidine with SAL or the co-administration of SAL with 0.5% lidocaine did not diminish the increased number of Fos-ir cells in TNC (Figure 5B,C,E). Conversely, the co-administration of 3 μg/kg of dexmedetomidine with 0.5% lidocaine significantly decreased the formalin-induced increase in Fos expression in ipsilateral TNC as compared with that in the SAL + SAL and DEX 3 + SAL groups (Figure 5D,E).

### 3.6. Co-Administration of Dexmedetomidine with 0.5% Lidocaine Does Not Affect Motor Performance, Blood Pressure, and Heart Rate

**Figure 6:**
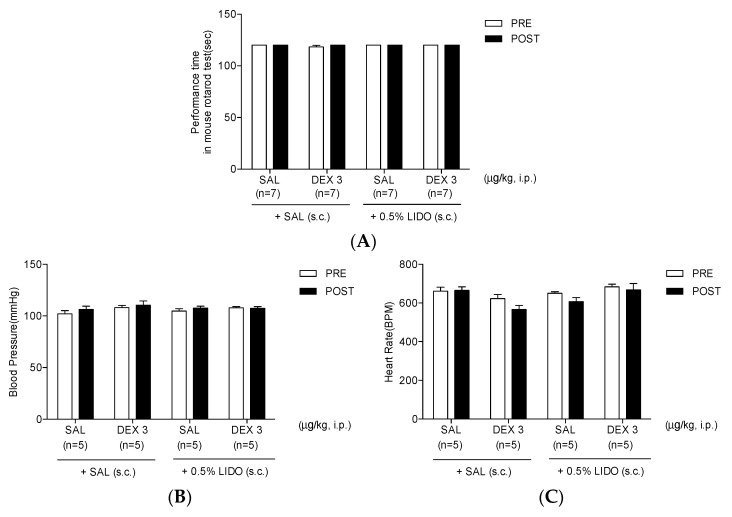
Effect of co-administration 0.5% lidocaine with dexmedetomidine (DEX) on motor coordination and systolic blood pressure. Neither the co-administration of 0.5% lidocaine with saline (SAL) nor low-dose of DEX (3 μg/kg) injection impact (**A**) motor coordination and (**B**,**C**) systolic blood pressure (as compared with that in the SAL + SAL group, *n* = 7 per group).

In the rotarod test, SAL or 3 μg/kg of dexmedetomidine with s.c. injection of SAL did not affect motor coordination (SAL + SAL or DEX 3 + SAL). SAL or 3 μg/kg of dexmedetomidine combined with 0.5% lidocaine also did not alter the motor performance time (Figure 6A). Moreover, no fall in the rotarod has been observed in all treated mice (data not shown). Similarly, in the SBP test, SAL or 3 μg/kg of dexmedetomidine with s.c. injection of SAL did not affect the normal blood pressure and heart rate (Figure 6B,C). Furthermore, neither the co-administration of SAL nor 3 μg/kg of dexmedetomidine with 0.5% lidocaine lessened the SBP and heart rate (Figure 6B,C).

## 4. Discussion

The study findings have shown that the administration of dexmedetomidine i.p. induced antinociceptive effects during the second phase of the orofacial formalin test in mice (Figure 1). Additionally, the number of Fos-ir cells in the ipsilateral TNC was reduced in the dexmedetomidine-treated mice as compared with those in the SAL-treated group (Figure 2). Dexmedetomidine, a highly specific α2-adrenergic receptor agonist, is mainly used clinically as an anxiolytic or sedative drug. However, the activation of α2-adrenoceptors in peripheral tissue or spinal cord has also been recognized to mediate the antinociceptive action of dexmedetomidine under several pain conditions [25]. In this regard, dexmedetomidine was reported to trigger antinociceptive effects in several animal models [26,27,28]. In our previous study, the time-dependent anti-allodynic effects of dexmedetomidine or clonidine, another α2-adrenergic receptor agonist, were also analyzed in a spared nerve injury mouse model [29]. Nevertheless, whether dexmedetomidine could reduce acute nociception rather than the sedative effects in the orofacial region was unclear; hence, this study was the first to show that dexmedetomidine administration developed antinociceptive effects in a mouse orofacial formalin model.

Generally, the activation of α2-adrenoceptors is correlated with a decrease in excitatory neurotransmitters at the central afferent terminals or induction of hyperpolarization in the spinal dorsal horn neurons through an increase in potassium conductance [25,30]. A recent study also showed that dexmedetomidine inhibited voltage-gated sodium channels in small-sized trigeminal ganglion neurons, by mediating α2-adrenoceptor activation [31]. Additionally, it was reported that dexmedetomidine produced a neuroprotective effect on the nervous system via anti-inflammatory, anti-excitotoxicity, and anti-oxidative actions, as well as the inhibition of neuronal apoptosis [25]. Jang et al. showed that i.p. injection of dexmedetomidine induced a significant antinociceptive effect, which might be associated with the attenuation of splenic natural killer (NK) cell activation by formalin injection [32]. However, they also found that the proliferative response of the lymphocytes or the production of cytokines, such as tumor necrosis factor- α (TNF- α) and interleukin 1β (IL-1β), was not affected by i.p. dexmedetomidine injection (30 μg/kg) [32]. On the other hand, Meng et al. demonstrated that dexmedetomidine (300 μg/kg) exhibited an analgesic effect on 2,4,6-Trinitrobenzenesulfonic acid (TNBS)-induced chronic inflammatory visceral pain in rats, which resulted in reduced pro-inflammatory cytokines via the miR-34a-mediated histone deacetylase 2 (HDAC2) pathway [33]. Moreover, it was also reported that dexmedetomidine (25 μg/kg) attenuated lipopolysaccharide (LPS)-induced acute lung injury and increased inflammatory cytokines in mice [34]. In these studies, although dexmedetomidine was reported to reduce inflammatory responses, including increased cytokine concentrations, it is important that the sedative doses of dexmedetomidine (25–300 μg/kg) were used. In this regard, further studies are needed to determine whether low or medium doses of dexmedetomidine may produce anti-inflammatory effects in an animal model of orofacial inflammatory pain.

In addition, dexmedetomidine can suppress peripheral formalin stimulus-induced increases of Fos-protein expression in TNC cells together with an antinociceptive effect. There was a strong correlation between the Fos-protein expression in the TNC cells and the intensity of nociceptive stimulation [35]. Since the trigeminal nervous system is activated by calcitonin gene-related peptide, bradykinin, and substance P of the orofacial or meningeal region, Fos-protein expression has been utilized as a sign of the functional activity of neurons in the TNC [36,37]. The study findings have shown that orofacial formalin-induced increase of Fos-ir cells in the TNC was significantly diminished in dexmedetomidine-treated mice, implying that the orofacial antinociceptive effect of dexmedetomidine was closely related to modulation of neuronal activity in TNC (e.g., the inhibition of central sensitization).

As shown in Figure 1, although 30 μg/kg of dexmedetomidine seemed to strongly suppress the nociceptive responses in both phases, its effect was sedative. During the second phase, no spontaneous nociceptive reaction was observed. In this regard, the effect of high-dose dexmedetomidine (30 μg/kg) was sedative, but not antinociceptive, in the formalin test. Therefore, the effects of dexmedetomidine on motor coordination have been subsequently analyzed using the rotarod test. The rotarod test has been commonly used to evaluate the motor coordination in animals in relation to the sedative action of experimental substances [38,39,40,41]. In our previous studies, we have also performed a rotarod test to investigate the sedative effect of α2 adrenoceptor-related drugs (e.g., clonidine, dexmedetomidine or p38 mitogen-activated protein kinase inhibitor) on motor coordination in animals with neuropathic pain [29,42,43]. Similarly, only animals that received high-dose dexmedetomidine (30 μg/kg) showed a decline in performance time and a rise in the number of falls on the rotating rotarod, respectively (Figure 3A,B). These results showed that the powerful effects of high-dose dexmedetomidine are triggered by the impaired motor function associated with the sedative effect. Moreover, high-dose dexmedetomidine has been well established to be typically accompanied by significant adverse effects that include hypotension, bradycardia, lethargy, and weakness [44,45]. The most common side effects of dexmedetomidine include hypotension and bradycardia. Therefore, the effects of multiple doses of dexmedetomidine on side effects, including SBP and bradycardia, were investigated using a noninvasive computerized tail-cuff system. Interestingly, only mice treated with high-dose dexmedetomidine (30 μg/kg) had significant declines in SBP and heart rate, whereas the lower doses of dexmedetomidine (3 and 10 μg/kg) did not affect either the blood pressure or heart rate. These findings demonstrate the dose-limiting effect of dexmedetomidine treatment on analgesic action in clinical applications.

It is important to address whether the anti-nociceptive effect of medium dose of dexmedetomidine was due to a sedative action. Several studies in the literature have reported that similar doses of dexmedetomidine might produce anti-nociceptive effect in various animal models [46,47]. Especially, Martin et al. found that 10 μg/kg of dexmedetomidine inhibited aggressive behaviors without a sedative effect [48]. In the present study, a medium dose of dexmedetomidine did not appear to affect the motor coordination in the rotarod test. Moreover, data from the immunohistochemistry for c-Fos protein, a biomarker of nociceptive neuronal activation (Figure 2 and Figure 5), also indicated that the anti-nociceptive effect of a medium dose of dexmedetomidine was associated with neuronal modulation in the TNC or spinal dorsal horn, but not the sedative action in higher brain center.

Lidocaine, widely used in medicating plaster, is recommended as a topical analgesic of first-line therapy for topical, peripheral, and neuropathic pain [49]. In this regard, Ralf et al. provided clinical evidence supporting the use of lidocaine in international guidelines for the treatment of localized neuropathic pain [50]. Moreover, Jeff et al. reported that lidocaine patches are recommended as first-line and second-line treatments for postherpetic neuralgia, and many institutions have published guidelines for clinical practice [51]. In recent years, lidocaine has been approved for intravenous use in clinical practice and is recognized as a safe agent to relieve various pain symptoms [52]. Many studies have also reported not only the effect of lidocaine alone on local anesthetic or analgesic potency but also its synergic or additive effect combined with other analgesics in different animal models or clinical trials [52,53,54,55,56]. Notably, several papers have shown that a low concentration of lidocaine (0.5%) and its derivative (QX-572) decreased formalin-induced nociceptive responses during the first phase, but not during the second phase [55,57]. This study also found that s.c. injection of 0.5% lidocaine alone significantly diminished the orofacial nociceptive response during the first phase (Figure 4B,C). Conversely, intrathecally or intraperitoneally applied lidocaine produced suppression of activity for both phases in the rat or mouse paw formalin test, respectively [54,58]. This discrepancy is believed to be caused by differences in the route of application or the concentration of lidocaine.

Although s.c. injection of 0.5% lidocaine did not reduce the second phase orofacial nociception and did not modify the rise in Fos-ir neurons in the TNC area, 0.5% lidocaine was believed to be a safe local anesthetic without side effects including itching, rash, swelling, or irritation. Therefore, the potential effects of the combination treatment of 0.5% lidocaine with ineffective low-dose dexmedetomidine (3 μg/kg) have been subsequently investigated. Interestingly, the co-administration of 0.5% lidocaine with an ineffective lower dose of dexmedetomidine caused an antinociceptive effect in both phases (Figure 4) as well as a significant drop in Fos-ir cells in the TNC region (Figure 5). Furthermore, whether the administration of 0.5% lidocaine alone or in combination with a low-dose of dexmedetomidine had effects on motor coordination, blood pressure, and heart rate, respectively, has been explored. Subcutaneous injection of 0.5% lidocaine alone did not affect the duration of the rotarod test, blood pressure, and heart rate. Similarly, co-administration of ineffective low-dose of dexmedetomidine and 0.5% lidocaine did not affect motor coordination nor the cardiovascular system Figure 6). Recently, in a randomized controlled trial study, Yamane et al. showed that co-administration of dexmedetomidine and lidocaine considerably boosted the local anesthetic strength of lidocaine without any major influences on the cardiovascular system when locally injected into the oral mucosa [56]. Moreover, in dentistry, the co-administration of dexmedetomidine and lidocaine blocked the maxillary and mandibular nerve and significantly prolonged the block duration and shortened the onset of action, as well as the need for fewer analgesics in the postoperative period [59,60].

The α2 adrenergic receptors in humans are widely distributed in the central nervous system (CNS), peripheral nervous system, autonomic ganglia, and other organ tissues. A few studies have shown that systemically administered α2 receptor agonist and dexmedetomidine produce anti-nociceptive effects in human and animal studies, which may be involved in neuronal modulation at the spinal and peripheral levels [51,61,62]. In this regard, Im et al. reported that inhibition of voltage-gated sodium channels in TG neurons, mediated by the activation of G-protein-coupled α2 adrenoceptors, might contribute to the analgesic effects of dexmedetomidine in the trigeminal system [31]. Taken together, we considered that the mechanism underlying the effect of systemically injected dexmedetomidine might involve the action of locally applied dexmedetomidine. In the present study, the route of administration of dexmedetomidine was systemic injection, and co-administration of 0.5% lidocaine with the ineffective lower dose of dexmedetomidine (3 μg/kg) significantly reduced the increase of Fos-ir cells in the ipsilateral TNC. Thus, it is reasonable that the antinociceptive effect of co-administration of 0.5% lidocaine with the ineffective lower dose of dexmedetomidine may be associated with the underlying mechanism at CNS, but not the peripheral site. In the further study, we plan to verify these issues (i.e., the comparison between the effects of local dexmedetomidine versus systemic dexmedetomidine and the investigation of centrally mediated mechanism underlying anti-nociceptive effect of systemic dexmedetomidine injection). Clinically, these studies can demonstrate which route can be more effective and safer when low-dose dexmedetomidine and lidocaine are administered together during acute stomatitis, mucogingival surgery, or after surgery. In addition, it can be further determined whether the co-treatment of lower doses of dexmedetomidine and lidocaine induces analgesic effect by reducing acute inflammatory reaction (e.g., decreased production of inflammatory cytokines or suppression of downstream signaling). Finally, the present study also suggests that co-administration of low-dose dexmedetomidine with a low concentration of lidocaine reduces both acute and inflammatory pain in the orofacial region, which may be a safe therapeutic strategy for OFP management without adverse effects including motor dysfunction, hypotension, and bradycardia.

## 5. Conclusions

This study has shown that dexmedetomidine significantly reduced nociceptive responses and TNC Fos expression in an orofacial formalin-induced pain model. Moreover, the co-administration of systemic low-dose dexmedetomidine and 0.5% lidocaine produced a strong antinociceptive effect, without causing any movement disorders, hypotension, or bradycardia. Altogether, the findings suggest that the combination therapy of low-dose of systemic dexmedetomidine with lidocaine might be safe and effective for the management of acute inflammatory pain in the orofacial region, such as mucogingival surgery or acute stomatitis.

## Figures and Tables

**Table 1:** Effect of dexmedetomidine (DEX) in the orofacial formalin-induced pain model.

Treatment	First Phase (s)	*p*-Value	Second Phase (s)	*p*-Value
SAL	37.77 ± 6.49	NA	436.36 ± 36.81	NA
DEX 3	44.43 ± 4.3	0.9989	415.34 ± 39.51	0.9805
DEX 10	38.49 ± 10.49	0.9176	195.20 ± 80.15 **	0.0045
DEX 30	1.23 ± 0.75 **	0.0018	0 ± 0 ***	0.0001

Notes: All values are mean ± S.E.M using one-way ANOVA followed by bonferroni test; ** *p* < 0.01 and *** *p* < 0.001. Abbreviations: ANOVA, analysis of variance; NA, not applicable; S.E.M, standard error of the mean.

**Table 2:** Effect of co-administration of 0.5% lidocaine with saline (SAL) or dexmedetomidine (DEX) in the orofacial formalin-induced pain model.

Treatment	First Phase (s)	*p*-Value	Second Phase (s)	*p*-Value
SAL + SAL	44.54 ± 4.22	NA	486.32 ± 47.44	NA
DEX 3 + SAL	45.35 ± 4.85	0.7067	495.05 ± 52.61	0.9999
SAL + LIDO	17.47 ± 2.97 **, ^##^	** 0.0064, ^##^ 0.0011	477.77 ± 79.28	0.9935
DEX 3 + LIDO	14.62 ± 3.09 **, ^###^	** 0.0041, ^###^ 0.0007	232.88 ± 26.20 **, ^##^	** 0.0043, ^##^ 0.0076

Notes: All values are mean ± S.E.M using one-way ANOVA followed by bonferroni test; ** *p* < 0.01 as compared with SAL + SAL. ^##^ *p* < 0.01, ^###^ *p* < 0.001 as compared with DEX3 + SAL. Abbreviations: ANOVA, analysis of variance; NA, not applicable; SEM, standard error of the mean.

## Data Availability

All the data in support of the findings presented are included and can be available by the corresponding author with any request.
